# Is Self-Rated Health Different for Nonagenarians?

**DOI:** 10.1093/geroni/igaf122.084

**Published:** 2025-12-31

**Authors:** Marja Jylha, Katariina Tuominen, Linda Enroth

**Affiliations:** Tampere University, Tampere, Pirkanmaa, Finland; Tampere University, Tampere, Pirkanmaa, Finland; Tampere University, Tampere, Pirkanmaa, Finland

## Abstract

Self-rated health (SRH) is a well-known predictor of mortality. In younger old people, chronic conditions are a major determinant of SRH. Less is known about SRH among people aged 90 and older, a small but rapidly growing population group with rather short life-expectancy and high multimorbidity. This paper discusses the contents and meanings of ´health´ in self-ratings of nonagenarians, contrasting different types of data from the Vitality 90+ project in Tampere, Finland. The project includes repeated survey studies with all individuals aged 90+ in the area plus qualitative interviews and face-to-face health examinations with subgroups. In the 2023 qualitative life story interviews (N = 54), participants framed diseases as given facts in high age and discussed health in relation to the ability to do things, manage everyday life, experienced symptoms, cognitive capacity and medications. They used comparisons to age peers with poorer condition or already deceased, to construct a relatively positive view of their own health. This is consistent with our earlier analysis in the survey data, where mobility and subjective symptoms were more important direct associates of SRH than diseases. Perhaps surprisingly, SRH showed a robust, graded association with mortality also in nonagenarians. During 4.6 years, mortality rate was 43% for good & fairly good and 89% for fairly poor & poor SRH. Our findings invite discussion about the meaning of ´health´ and about the most relevant experienced hallmarks of physiological vitality in the oldest-old.

